# Association of Surgeon Case Numbers of Pancreaticoduodenectomies vs Related Procedures With Patient Outcomes to Inform Volume-Based Credentialing

**DOI:** 10.1001/jamanetworkopen.2020.3850

**Published:** 2020-04-29

**Authors:** Kyle H. Sheetz, Usha Nuliyalu, Hari Nathan, Christopher J. Sonnenday

**Affiliations:** 1 Department of Surgery, University of Michigan, Ann Arbor; 2 Center for Healthcare Outcomes and Policy, School of Medicine, University of Michigan, Ann Arbor

## Abstract

**Importance:**

Despite growing interest from various surgical societies and patient safety organizations, concerns remain that volume-based credentialing standards are arbitrary and may fail to recognize a surgeon’s full scope of practice.

**Objective:**

To evaluate whether surgeon experience with related procedures was associated with better outcomes for pancreaticoduodenectomy compared with procedure-specific experience alone.

**Design, Setting, and Participants:**

This proof-of-concept cohort study used the all-payer State Inpatient Databases from 6 geographically diverse states to identify all operations for surgeons who performed at least 1 pancreaticoduodenectomy from January 1, 2012, to December 31, 2014. Each surgeon’s mean annual volume for pancreaticoduodenectomies and related complex hepatopancreatobiliary (HPB) procedures was calculated. Outcomes for surgeons above and below a threshold of 12 pancreaticoduodenectomies per year were evaluated. Whether related HPB procedure volume was also associated with better outcomes for surgeons not meeting the procedure-specific threshold was also evaluated. Data were analyzed from March 2 through 20, 2019.

**Main Outcomes and Measures:**

Thirty-day mortality and complications.

**Results:**

The study cohort included 176 043 patients, of whom 92 064 were female (52.3%), with a mean (SD) age of 59 (17) years. Within 270 hospitals, only 54 of 1028 surgeons (5.3%) met the mean pancreaticoduodenectomy volume threshold from 2012 to 2014. In-hospital mortality after pancreaticoduodenectomy was lower for surgeons who performed 12 or more procedures per year (1.8% [95% CI, 1.1%- 2.4%] vs 4.7% [95% CI, 4.0%-5.4%]; odds ratio, 0.32; 95% CI, 0.21-0.50). However, in-hospital mortality varied 7-fold among surgeons who did not meet the threshold (1.2% [95% CI, 0.8%-1.6%] to 8.4% [95% CI, 7.9%-8.9%]). Increasing HPB case volume was associated with better outcomes for pancreaticoduodenectomy in this group. For example, surgeons performing 2 or fewer pancreaticoduodenectomies annually would need to perform an additional 27 related HPB procedures to match the in-hospital mortality rate of surgeons performing 12 or more pancreaticoduodenectomies.

**Conclusions and Relevance:**

In this proof-of-concept cohort study, few surgeons met even modest annual volume thresholds for pancreaticoduodenectomy. The findings suggest that inclusion of related procedure volumes may safely expand the cohort of surgeons credentialed to perform certain procedures under volume-based standards.

## Key Points

**Question:**

Is surgeon experience with related procedures associated with better outcomes for pancreaticoduodenectomy compared with procedure-specific experience alone?

**Findings:**

In this proof-of-concept cohort study of 176 043 patients and 1028 surgeons, 54 surgeons (5.3%) met modest annual volume thresholds for pancreaticoduodenectomy. However, increasing related hepatopancreatobiliary case volume was associated with better outcomes for pancreaticoduodenectomy.

**Meaning:**

These findings suggest that related procedure volumes may be used to inform surgeon-specific, volume-based credentialing standards.

## Teaser

This proof-of-concept cohort study evaluates whether surgeon experience with related procedures was associated with better outcomes for pancreaticoduodenectomy compared with procedure-specific experience alone.

## Introduction

Hospital credentialing remains an important process to ensure that surgeons have the appropriate experience to safely perform certain surgical procedures.^1^ Building on considerable evidence that higher surgeon volume is associated with better patient safety, various clinical societies and patient safety advocates contend that procedure-specific volume standards can improve the quality of the credentialing process.^2,3,4,5^ For example, the Leapfrog Group suggests that hospitals restrict pancreatic cancer resections to surgeons who perform a minimum of 10 such operations per year.^6^ Although these standards remain discretionary, some health systems are now limiting high-risk operations, such as pancreatectomy, to surgeons who meet prespecified volume standards.^7^

Despite the potential to improve patient safety, numerous concerns exist with implementing surgeon-specific volume credentialing standards. Surgeon-specific outcomes measurements may be unreliable. One study focusing on colectomy^8^ found that 86% of the variation in complication rates between surgeons was attributable to statistical noise, and only 1 surgeon throughout an entire state performed enough operations to achieve a reasonable threshold of reliability. Others argue that the implementation of surgeon-specific volume standards may exacerbate disparities because care is restricted to even fewer surgeons and centers.^9^ These standards may also ignore surgeons’ full scope of practice, where cumulative experience with related procedures is also associated with better outcomes for complex operations, such as esophagectomy.^10^ The feasibility of using surgeons’ full scope of practice to credential low-volume surgeons remains unclear.

To better understand how experience with other procedures may serve as a surrogate for credentialing surgeons to perform uncommon procedures, we examined the association between related procedure volume and short-term outcomes after pancreaticoduodenectomy. We used the State Inpatient Databases (SID) to capture the entire practice of all surgeons who performed at least 1 pancreaticoduodenectomy from January 1, 2012, through December 31, 2014. Among low-volume surgeons, we evaluated whether experience with related hepatopancreatobiliary (HPB) procedures was associated with better outcomes for pancreaticoduodenectomy compared with procedure-specific experience alone.

## Methods

### Data Source and Study Population

Owing to the use of retrospective data from the SID, this cohort study was deemed exempt from approval and informed consent by the institutional review board at the University of Michigan, Ann Arbor. This report followed the Strengthening the Reporting of Observational Studies in Epidemiology (STROBE) reporting guideline for cohort studies.

The SID is maintained as part of the Agency for Healthcare Research andQuality’s Hospital Cost and Utilization Project . We captured data from New York, Florida, Maryland, Washington, Michigan, and New Jersey. For these states, episode-level data are linked to discrete physician identifiers that permit associating cases with specific surgeons. These states were also chosen because they represent multiple geographic regions, varying population sizes, and varying health care markets.

We collected data on patient age, operative diagnosis, demographics, geographic location, and comorbidities. We identified patients undergoing pancreaticoduodenectomy using the *International Classification of Diseases, Ninth Revision, Clinical Modification* (*ICD-9-CM*), code 52.7. We identified a subset of procedures, not including cholecystectomy, deemed to be anatomically and technically related to pancreaticoduodenectomy using *ICD-9-CM* codes. A full list of related HPB procedures is included in the eMethods in the Supplement. We linked the SID files to the American Hospital Association Annual Survey to obtain additional information on hospital size, resources, and other characteristics.

### Outcomes

Our primary outcomes of interest were in-hospital death or the incidence of any postoperative complication within 30 days of surgery. We used *ICD-9-CM* codes to identify complications such as pulmonary failure, pneumonia, myocardial infarction, deep venous thrombosis/pulmonary embolism, renal failure, surgical site infection, gastrointestinal tract bleeding, and hemorrhage. These complications represent a subset of codes with the highest sensitivity and specificity.^11^ Because of the frequency in which pancreatic leak or fistula (*ICD-9-CM* code 577.8) occurs after pancreaticoduodenectomy, we also included this outcome as a complication.

### Statistical Analysis

Data were analyzed from March 2 through 20, 2019. The purpose of this analysis was to evaluate the association between postoperative outcomes and pancreaticoduodenectomy-specific case volume vs related HPB case volume at the surgeon level. All case volumes were summed, and the mean was calculated for each surgeon during the 3 years in the study. This process was used to avoid bias that may come from year-to-year variation in surgical volume.

We fit mixed-effects logistic regression models to adjust outcomes for differences in patient and hospital characteristics. All models included patient age, race, and 27 Elixhauser comorbidities used for risk adjustment with claims data.^12^ We further adjusted for hospital bed size, profit status, and nurse-to-patient ratios. We accounted for year-to-year differences in outcomes by including a dummy variable for each study year. Finally, we accounted for overall hospital factors by including a hospital identifier random-effect parameter. All estimates for a particular outcome were generated from a single model for each strata of surgeon volume. Robust CIs were generated from bootstrapping with 1000 replications made at the hospital level.

We report outcomes stratified by 3 volume thresholds (6, 12, and 36 procedures per year) to demonstrate the relative differences in outcomes as surgeon volume increases. These thresholds were chosen to demonstrate a concept, not to reflect ideal or specific annual volume thresholds. They were also chosen because they reflect intuitive annual volumes (eg, 12 per year equals 1 pancreaticoduodenectomy per month). For each threshold, surgeon volume categories were included in the primary models as a dichotomous variable. To determine the incremental effect of related HPB volume, we included this continuous variable in the model described above. We then estimated the relative effect of each per-case increase from the coefficients derived from this variable in each model. These results were stratified by surgeons performing fewer than 12 pancreaticoduodenectomies annually into quartiles (<1 to 2, bottom 25%; 3-5, middle 50%; and 6-11, top 25%). To test whether overall hospital volume influenced our main estimates, we repeated this analysis accounting for annual hospital volume as a continuous variable. We chose to model hospital volume continuously to avoid creating potentially arbitrary volume cutoffs that may be unique to our data source and thus less generalizable.

All statistical analyses were performed using STATA statistical software, version 14.2 (StataCorp LLC). We used a 2-sided approach at the 5% significance level for all hypothesis testing.

## Results

The characteristics of patients, surgeons, and hospitals are reported in Table 1. The mean (SD) age of the 176 043 patients was 59 (17) years; 83 979 were male (47.7%) and 92 064 were female (52.3%). Patients had a mean (SD) of 2 (2) comorbidities, and Medicare was the most common primary insurance (81 766 patients [46.4%]). Two hundred seventy hospitals were included in the study. Hospitals performed a median of 15 (interquartile range [IQR], 1-34) pancreaticoduodenectomies per year and saw a median of 30 (IQR, 1-75) related HPB cases per year. Teaching hospitals represented 18.1% (n = 49) of the cohort.

**Table 1.** Patient, Surgeon, and Hospital Characteristics, 2012-2014

Characteristic	Data^a^
**Patients**	
No. of patients	176 043
Age, mean (SD), y	59 (17)
Male	83 979 (47.7)
White	108 175 (61.4)
African American	23 218 (13.2)
No. of comorbid conditions, mean (SD)	2 (2)
Private insurance	62 006 (35.2)
Medicare	81 766 (46.4)
Medicaid	20 047 (11.4)
Pancreaticoduodenectomy	7657 (4.3)
Related HPB procedures	17 168 (9.8)
**Surgeons**	
No. of surgeons	1028
Pancreaticoduodenectomy volume	
Mean (SD)	5 (10)
Median (IQR)	1 (1-6)
Related HPB volume	
Mean (SD)	13 (21)
Median (IQR)	2 (0-18)
**Hospitals**	
No. of hospitals	270
Pancreaticoduodenectomy volume	
Mean (SD)	63 (102)
Median (IQR)	15 (1-34)
Related HPB volume	
Mean (SD)	124 (201)
Median (IQR)	30 (1-75)
Bed size	
<200	70 (25.9)
200-349	81 (30.0)
350-499	58 (21.5)
≥500	61 (22.6)
Rural	8 (3.0)
Teaching	49 (18.1)
Nurse-to-patient ratio, mean (SD)	1.8 (1)

Abbreviations: HPB, hepatopancreatobiliary; IQR, interquartile range.

^a^ Unless otherwise indicated, data are expressed as number (percentage) of patients.

The 1028 surgeons included in the study performed a total of 7657 pancreaticoduodenectomies from January 2012 through the end of December 2014. The median annual surgeon volume of pancreaticoduodenectomy was 1 case per year (IQR, 1-6). Eight hundred forty-three surgeons (82.0%) performed a mean of 2 or fewer pancreaticoduodenectomies during the study period. The median annual volume of related HPB procedures was 2 (IQR, 0-18). The most common operations included in the 17 168 related HPB procedures included partial hepatectomy (3926 [22.9%]), distal pancreatectomy (2825 [16.5%]), and hepatic lobectomy (1456 [8.5%]) (Table 2). A dot plot representation of the distribution of pancreaticoduodenectomy and related HPB procedure volume is included in eFigure 1 in the Supplement.

**Table 2.** Description of Most Common Related Hepatopancreatobiliary Procedures

Procedure	*ICD-9-CM* procedure code	No. (%) of procedures^a^
Partial hepatectomy	50.22	3926 (22.9)
Distal pancreatectomy	52.52	2825 (16.5)
Hepatic lobectomy	50.3	1456 (8.5)
Liver transplant	50.5	926 (5.4)
Biliary reconstruction	51.37	585 (3.4)
Total pancreatectomy	52.6	465 (2.7)
Other pancreatectomy	52.59	429 (2.5)
Other destruction of liver lesion	50.29	413 (2.4)
Other excision of pancreas or pancreatic duct	50.29	413 (2.4)
Laparoscopic ablation of liver lesion	50.25	370 (2.2)

Abbreviation: *ICD-9-CM*, *International Classification of Diseases, Ninth Revision, Clinical Modification*.

^a^ Includes 17 168 procedures.

A small proportion of surgeons met each volume threshold (Table 3). For example, only 54 surgeons (5.3%) performed more than 12 pancreaticoduodenectomies per year. Table 3 also shows differences in outcomes for surgeons grouped by each volume threshold. In-hospital mortality rates were 4.7% (95% CI, 4.0%-5.4%) for surgeons who performed fewer than 12 pancreaticoduodenectomies annually compared with 1.8% (95% CI, 1.1%-2.4%) for surgeons performing more than 12 (odds ratio [OR], 0.32; 95% CI, 0.21-0.50). The difference in mortality rates between low-volume and high-volume surgeons was only slightly smaller after accounting for hospital volume (1.9% [95% CI, 1.2%-2.6%] vs 4.4% [95% CI, 3.5%-5.2%]; OR, 0.39; 95% CI, 0.23- 0.66). Complications were also significantly lower for surgeons exceeding the threshold of 12 pancreaticoduodenectomies per year (30.4% [95% CI, 27.4%-33.4%] vs 35.8% [95% CI, 33.3%-38.1%]; OR, 0.75; 95% CI, 0.63- 0.91).

**Table 3.** Risk-Adjusted Complications and In-Hospital Mortality Stratified by Surgeon Volume for Pancreaticoduodenectomy

Volume threshold^a^	Risk-adjusted rates (95% CI), %		Odds ratio (95% CI)
	Surgeons below threshold	Surgeons above threshold	
In-hospital mortality			
6 Per year (102 surgeons [9.9%])	4.9 (4.1-5.7)	2.2 (1.6-2.9)	0.41 (0.27-0.61)
Accounting for hospital volume	4.3 (3.5-5.3)	2.5 (1.8-3.2)	0.52 (0.34-0.79)
12 Per year (54 surgeons [5.3%])	4.7 (4.0-5.4)	1.8 (1.1-2.4)	0.32 (0.21-0.50)
Accounting for hospital volume	4.4 (3.5-5.2)	1.9 (1.2-2.6)	0.39 (0.23-0.66)
36 Per year (10 surgeons [1.0%])	3.5 (3.0-4.1)	2.2 (1.2-3.2)	0.57 (0.34-0.97)
Accounting for hospital volume	3.3 (2.7-3.9)	3.0 (1.9-4.2)	0.89 (0.51-1.56)
Complications			
6 Per year (102 surgeons [9.9%])	38.5 (35.8-41.1)	30.4 (27.9-32.8)	0.66 (0.57-0.78)
Accounting for hospital volume	39.0 (35.7-42.3)	30.1 (27.8-32.5)	0.64 (0.53-0.77)
12 Per year (54 surgeons [5.3%])	35.8 (33.3-38.1)	30.4 (27.4-33.4)	0.75 (0.63-0.91)
Accounting for hospital volume	36.2 (33.3-39.0)	30.1 (27.2-33.0)	0.73 (0.60-0.90)
36 Per year (10 surgeons [1.0%])	33.8 (31.8-35.8)	29.1 (23.9-34.3)	0.78 (0.59-1.03)
Accounting for hospital volume	33.9 (31.8-35.9)	28.8 (24.3-33.2)	0.76 (0.60-0.96)

^a^ Derived from 3-year mean pancreaticoduodenectomy volume. The low-volume category is baseline for all comparisons. The thresholds of 6, 12, and 36 procedures per year are meant to be examples of potential volume thresholds rather than reflecting ideal or specific volumes to achieve a desired outcome. Hospital volume was modeled continuously in analyses also accounting for hospitals’ annual experience with pancreaticoduodenectomy. C statistics for all models ranged from 0.74 to 0.87.

In-hospital mortality varied 7-fold (1.2% [95% CI, 0.8%-1.6%] to 8.4% [95% CI, 7.9%-8.9%]) and complications varied 2-fold (28% [95% CI, 26%-30%] to 56% [95% CI, 51%-61%]) among surgeons who did not meet the 12-case threshold. Among low-volume pancreaticoduodenectomy surgeons, mortality and complications varied significantly with nonpancreaticoduodenectomy HPB case volume. Figure, A, shows the estimated number of additional related HPB procedures surgeons would need to perform to match the postoperative mortality rates of surgeons performing 12 or more pancreaticoduodenectomies per year. For the lowest-volume surgeons (1-2 pancreaticoduodenectomies annually), increasing related HPB case volume was associated with lower postoperative mortality. For example, those surgeons would need to perform an estimated additional 27 related HPB procedures to match the in-hospital mortality rate of surgeons performing 12 pancreaticoduodenectomies annually (Figure, A). Moderate-volume surgeons (6-11 pancreaticoduodenectomies annually) would need to perform an additional 10 related HPB procedures to match the complication rate of surgeons performing 12 or more (Figure, B).

**Figure.**
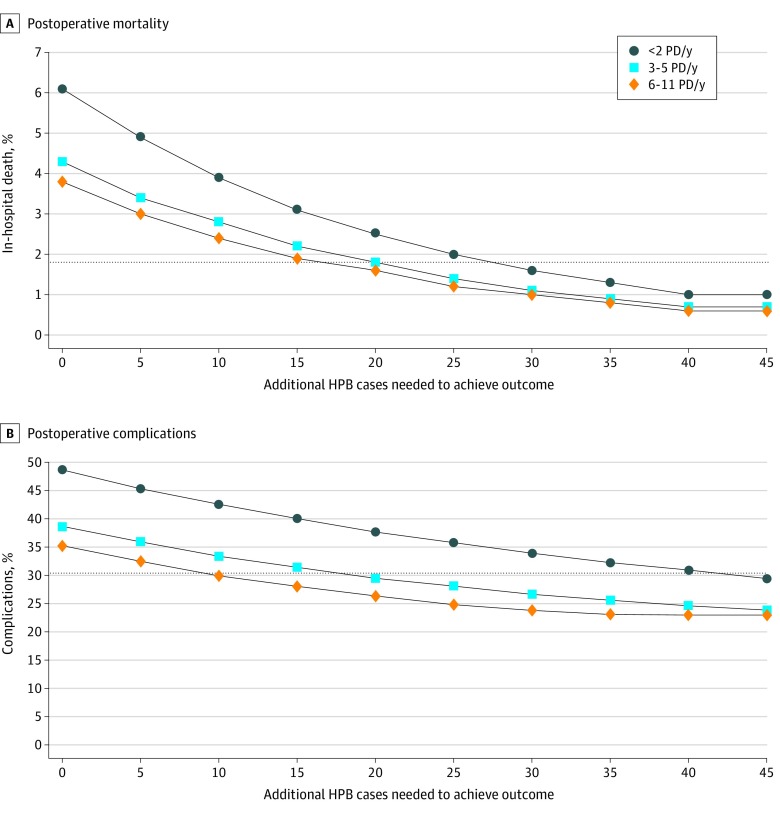
Postoperative Mortality and Complications Estimates of the number of additional hepatopancreatobiliary (HPB) cases required to achieve similar estimated postoperative mortality rates (A) and postoperative complication rates (B) for surgeons who perform 12 or more pancreaticoduodenectomies (PD) per year. Results are stratified by surgeon volume quartile. The middle 50% of surgeons are collapsed into 1 group. Horizontal lines indicate the threshold of 1.8% for in-hospital deaths at a volume of 12 PD/y and 30.4% for complications at a volume of 12 PD/y.

Adjusting for hospital volume did not significantly change these associations for surgeons performing 3 to 5 or 6 to 11 pancreaticoduodenectomies per year (eFigure 2 in the Supplement). However, after adjusting for hospital volume, estimates for surgeons performing 1 to 2 pancreaticoduodenectomies per year never matched the outcomes of surgeons performing 12 or more pancreaticoduodenectomies.

## Discussion

In this population-based study of all-payer claims data from 6 large states, we found that most of the pancreaticoduodenectomies are performed by surgeons who perform fewer than 2 per year. Higher surgical volume was associated with better outcomes. Among surgeons performing a volume threshold of 12 procedures per year, we observed 7-fold variation in mortality and 2-fold variation in complications. Related HPB surgical volume was associated with better outcomes after pancreaticoduodenectomy. For groups of moderate-volume surgeons (3-5 and 6-11 cases per year), higher related HPB volume was associated with better outcomes for pancreaticoduodenectomy. These results suggest that, for moderate-volume pancreaticoduodenectomy surgeons, related HPB volume may be a metric that can be used to differentiate surgeons with respect to pancreaticoduodenectomy outcomes. However, for the lowest volume surgeons (≤2 pancreaticoduodenectomies per year), higher related HPB volume was not reliably associated with better outcomes for pancreaticoduodenectomy, especially after accounting for hospital volume. This finding suggests that related HPB case volume may not be an effective way to reliably differentiate surgeons who perform a very low volume (1-2 per year) of pancreaticoduodenectomies.

The American Board of Medical Specialties and American Board of Surgery are interested in establishing standards for practicing physicians that provide information to patients, hospitals, and credentialing bodies that identify physicians with expertise in a particular domain. This process is particularly relevant in specialties with no formal board certification.^13^ For surgical subspecialties, accurate data about surgeons’ clinical practice in the “real world” and its association with patient outcomes are needed to inform the construction of sensible criteria for a focused practice designation in HPB surgery. Our data suggest that basing these distinctions on procedure-specific experience may exclude surgeons who would otherwise be able to safely perform necessary and lifesaving operations. Using related procedure volume may therefore be a strategy for clinical societies and credentialing bodies to balance the inherent tensions between quality and patient access.

A considerable amount of research already exists on the association between higher surgeon or hospital volume and better outcomes after pancreaticoduodenectomy.^2,4,5^ These studies have been used by surgical societies and patient safety organizations to advocate for minimum volume standards and greater centralization of high-risk operations.^4,6,14,15,16^ Others have raised concerns that minimum volume standards for specific procedures may exclude surgeons who are otherwise able to perform safe operations. For example, a recent study found that higher surrogate procedure volumes were associated with better outcomes after esophagectomy. As a result, current volume standards for pancreaticoduodenectomy (and other high-risk operations) may fail to acknowledge the entirety of an individual surgeon’s practice and experience. Thus, there is utility in understanding whether surgeons who perform procedures with technical similarities to an index procedure have better outcomes than surgeons whose practice is much less specific.

This study expands on prior work in several ways. We use the all-payer SID to capture the full scope of surgeons’ practices during a 3-year period. This scope is not possible with the National Inpatient Sample, which has been used in similar studies and reflects only a sample of cases from hospitals and surgeons. We also estimate the extent to which increases in related procedure volume were associated with better outcomes for pancreaticoduodenectomy. This estimate has direct relevance to the current discussion of differences in training pathways to HPB surgery and its effect on surgeon expertise and subsequent patient outcomes. Understanding how case mix in practice affects outcomes for index procedures may inform collaborative efforts among training programs and societies toward defining appropriate training pathways and requirements.

### Limitations

The results of this study should be interpreted within the context of certain limitations. Our data do not contain information that influences case complexity (eg, tumor size or prior operations) and therefore may limit our ability to account for all aspects of a case that may influence outcomes. We also lack information on surgeons’ background, prior experience, and training. That said, we capture a full 3 years of their current practice and base our analysis on volume standards that are currently the most common method to differentiate clinicians for surgical practice standards. Our analysis was not designed to determine the “correct” volume or a threshold volume required to perform a safe pancreaticoduodenectomy. However, we demonstrate outcomes with respect to standards at certain prespecified annual volumes. The purpose of this method is to demonstrate what tradeoffs (ie, access vs safety, or feasibility vs ability to differentiate surgeons) may need to be reflected in discussions around volume-based credentialing rather than to be prescriptive about discrete standards themselves.

## Conclusions

In this cohort study, we found that few surgeons performed an annual volume of pancreaticoduodenectomies to meet even modest volume standards. A higher surgeon volume of related HPB procedures was associated with better outcomes for pancreaticoduodenectomy. Leveraging these related procedural volumes may improve procedure-based credentialing standards and better reflect the full scope of surgeons’ practices, especially for surgeons who perform a low volume of certain complex operations.

## Notes

Supplement. **eMethods.** Procedure Definitions **eFigure 1.** Dot Plot of Surgeon Annual Volumes **eFigure 2.** Estimates Accounting for Hospital Volume
