# Supplementary material for: Association of Surgeon Case Numbers of Pancreaticoduodenectomies vs Related Procedures With Patient Outcomes to Inform Volume-Based Credentialing
Source: JAMA Netw Open. 2020 Apr 29;3(4):e203850. doi: 10.1001/jamanetworkopen.2020.3850 (PMC7191322; doi:10.1001/jamanetworkopen.2020.3850)
Supplement: Supplement. — eMethods. Procedure Definitions eFigure 1. Dot Plot of Surgeon Annual Volumes eFigure 2. Estimates Accounting for Hospital Volume [file jamanetwopen-3-e203850-s001.pdf]

Supplementary Online Content

Sheetz KH, Nuliyalu U, Nathan H, Sonneday CJ. Association of surgeon case numbers of pancreaticoduodenectomies vs related procedures with patient outcomes to inform volume-based credentialing. *JAMA Netw Open*. 2020;3(4):e203850. doi:10.1001/jamanetworkopen.2020.3850

eMethods. Procedure Definitions

eFigure 1. Dot Plot of Surgeon Annual Volumes

eFigure 2. Estimates Accounting for Hospital Volume

This supplementary material has been provided by the authors to give readers additional information about their work.

©2020 Sheetz KH et al. *JAMA Network Open*.

eMethods. Procedure Definitions

50 Operations On Liver

50.2 Local Excision Or Destruction Of Liver Tissue Or Lesion
- 50.21 Marsupialization of lesion of liver convert 50.21 to ICD-10-PCS
- 50.22 Partial hepatectomy convert 50.22 to ICD-10-PCS
- 50.23 Open ablation of liver lesion or tissue convert 50.23 to ICD-10-PCS
- 50.24 Percutaneous ablation of liver lesion or tissue convert 50.24 to ICD-10-PCS
- 50.25 Laparoscopic ablation of liver lesion or tissue convert 50.25 to ICD-10-PCS
- 50.26 Other and unspecified ablation of liver lesion or tissue convert 50.26 to ICD-10-PCS
- 50.29 Other destruction of lesion of liver convert 50.29 to ICD-10-PCS

50.3 Lobectomy of liver convert 50.3 to ICD-10-PCS

50.4 Total hepatectomy convert 50.4 to ICD-10-PCS

50.5 Liver Transplant
- 50.51 Auxiliary liver transplant convert 50.51 to ICD-10-PCS
- 50.59 Other transplant of liver convert 50.59 to ICD-10-PCS

50.9 Other Operations On Liver
- 50.92 Extracorporeal hepatic assistance convert 50.92 to ICD-10-PCS
- 50.93 Localized perfusion of liver convert 50.93 to ICD-10-PCS

51 Operations On Gallbladder And Biliary Tract

51.3 Anastomosis Of Gallbladder Or Bile Duct
- 51.31 Anastomosis of gallbladder to hepatic ducts convert 51.31 to ICD-10-PCS
- 51.32 Anastomosis of gallbladder to intestine convert 51.32 to ICD-10-PCS
- 51.33 Anastomosis of gallbladder to pancreas convert 51.33 to ICD-10-PCS
- 51.34 Anastomosis of gallbladder to stomach convert 51.34 to ICD-10-PCS
- 51.35 Other gallbladder anastomosis convert 51.35 to ICD-10-PCS
- 51.36 Choledochoenterostomy convert 51.36 to ICD-10-PCS
- 51.37 Anastomosis of hepatic duct to gastrointestinal tract convert 51.37 to ICD-10-PCS
- 51.39 Other bile duct anastomosis convert 51.39 to ICD-10-PCS

51.6 Local Excision Or Destruction Of Lesion Or Tissue Of Biliary Ducts And Sphincter Of Oddi
- 51.62 Excision of ampulla of Vater (with reimplantation of common duct) convert 51.62 to ICD-10-PCS

51.7 Repair Of Bile Ducts
- 51.71 Simple suture of common bile duct convert 51.71 to ICD-10-PCS
- 51.72 Choledochoplasty convert 51.72 to ICD-10-PCS
- 51.79 Repair of other bile ducts convert 51.79 to ICD-10-PCS

51.8 Other Operations On Biliary Ducts And Sphincter Of Oddi
- 51.82 Pancreatic sphincterotomy convert 51.82 to ICD-10-PCS
- 51.83 Pancreatic sphincteroplasty convert 51.83 to ICD-10-PCS

51.9 Other Operations On Biliary Tract
- 51.93 Closure of other biliary fistula convert 51.93 to ICD-10-PCS
- 51.94 Revision of anastomosis of biliary tract convert 51.94 to ICD-10-PCS

©2020 Sheetz KH et al. *JAMA Network Open*.

52 Operations On Pancreas

52.0 Pancreatotomy
- 52.09 Other pancreatotomy convert 52.09 to ICD-10-PCS
- 52.22 Other excision or destruction of lesion or tissue of pancreas or pancreatic duct convert 52.22 to ICD-10-PCS

52.3 Marsupialization of pancreatic cyst convert 52.3 to ICD-10-PCS
52.4 Internal drainage of pancreatic cyst convert 52.4 to ICD-10-PCS
52.5 Partial Pancreatectomy
- 52.51 Proximal pancreatectomy convert 52.51 to ICD-10-PCS
- 52.52 Distal pancreatectomy convert 52.52 to ICD-10-PCS
- 52.53 Radical subtotal pancreatectomy convert 52.53 to ICD-10-PCS
- 52.59 Other partial pancreatectomy convert 52.59 to ICD-10-PCS

52.6 Total pancreatectomy convert 52.6 to ICD-10-PCS
52.7 Radical pancreaticoduodenectomy convert 52.7 to ICD-10-PCS
52.8 Transplant Of Pancreas
- 52.80 Pancreatic transplant, not otherwise specified convert 52.80 to ICD-10-PCS
- 52.81 Reimplantation of pancreatic tissue convert 52.81 to ICD-10-PCS
- 52.82 Homotransplant of pancreas convert 52.82 to ICD-10-PCS
- 52.83 Heterotransplant of pancreas convert 52.83 to ICD-10-PCS
- 52.84 Autotransplantation of cells of Islets of Langerhans convert 52.84 to ICD-10-PCS
- 52.85 Allotransplantation of cells of Islets of Langerhans convert 52.85 to ICD-10-PCS
- 52.86 Transplantation of cells of Islets of Langerhans, not otherwise specified convert 52.86 to ICD-10-PCS

52.9 Other Operations On Pancreas
- 52.95 Other repair of pancreas convert 52.95 to ICD-10-PCS
- 52.96 Anastomosis of pancreas convert 52.96 to ICD-10-PCS

©2020 Sheetz KH et al. *JAMA Network Open*.

eFigure 1. Dot Plot of Surgeon Annual Volumes
Note: Dots represent individual surgeons.

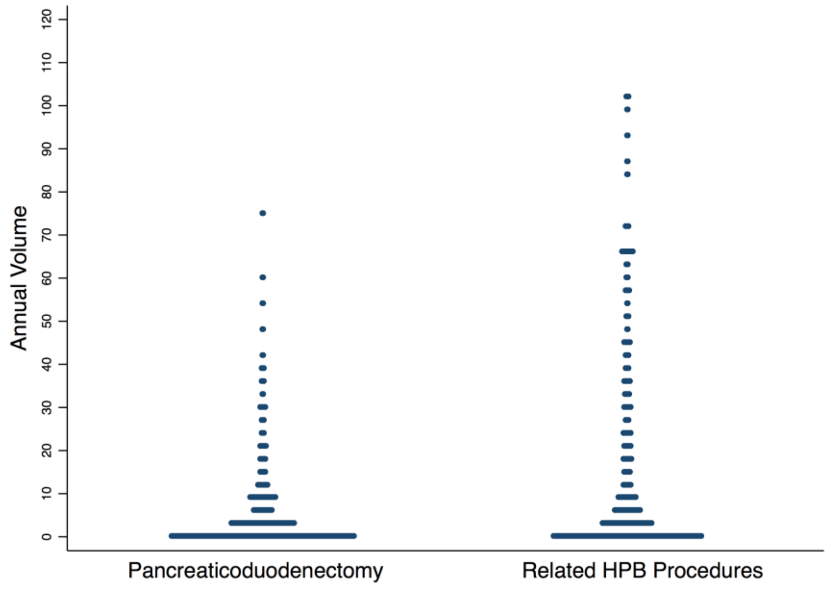

©2020 Sheetz KH et al. *JAMA Network Open*.

eFigure 2. Estimates Accounting for Hospital Volume
Note: Hospital volume was modeled as a continuous variable.

A – Postoperative Mortality

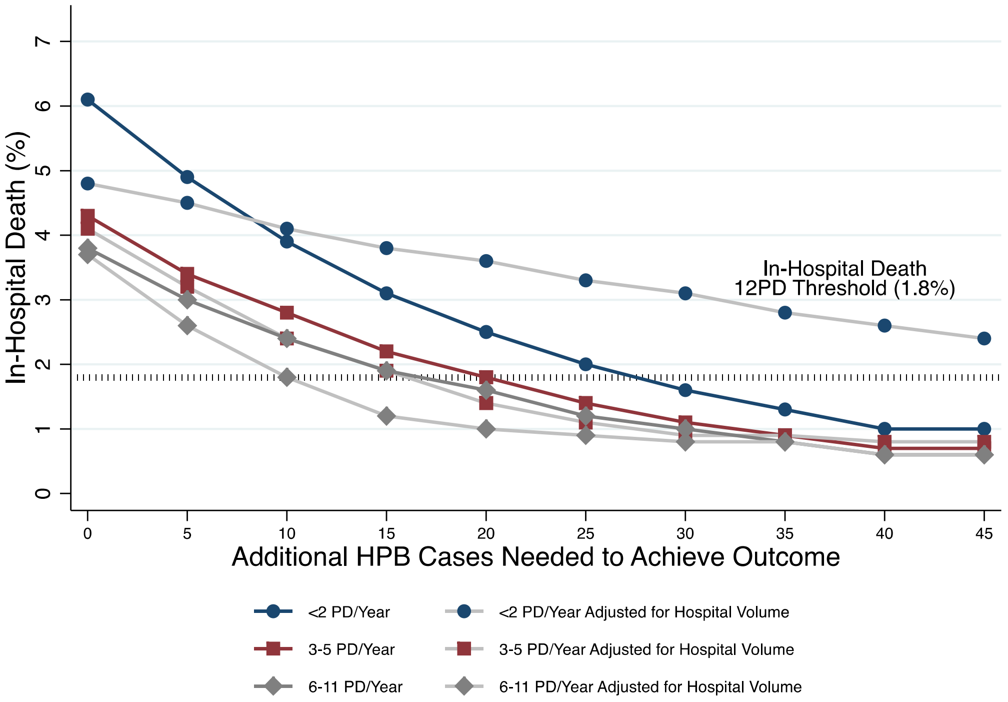

©2020 Sheetz KH et al. *JAMA Network Open*.

B – Postoperative Complications

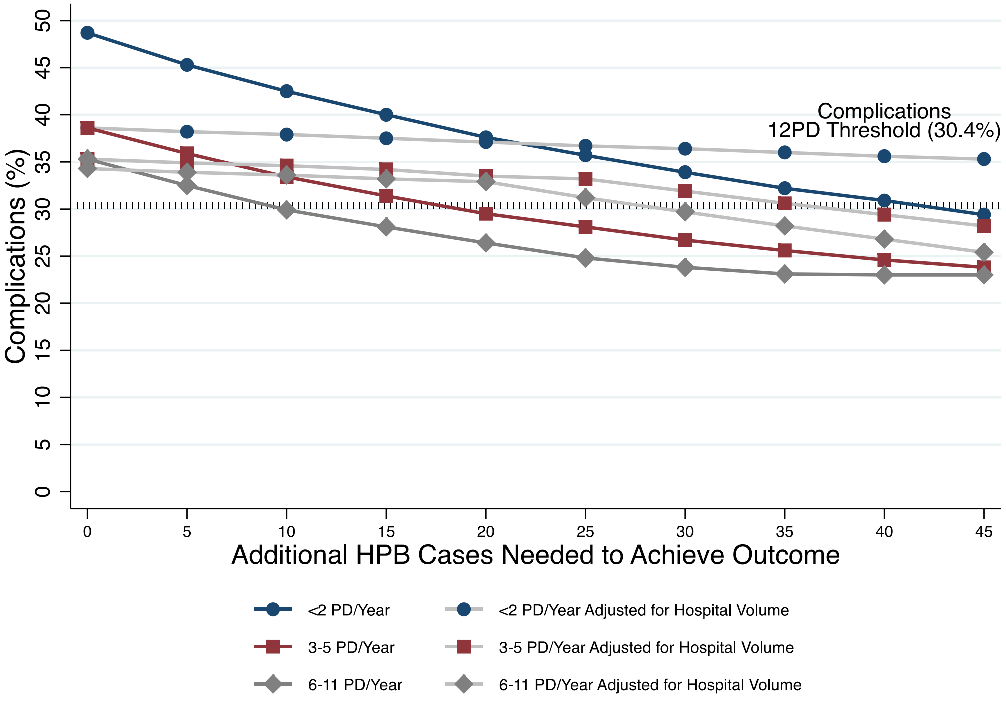

©2020 Sheetz KH et al. *JAMA Network Open*.
